# Changes in body mass index and waist circumference and heart failure in type 2 diabetes mellitus

**DOI:** 10.3389/fendo.2023.1305839

**Published:** 2023-12-21

**Authors:** Yang Zhou, Xiangping Chai, Guifang Yang, Xin Sun, Zhenhua Xing

**Affiliations:** ^1^Department of Critical Care Medicine, Second Xiangya Hospital, Central South University, Changsha, China; ^2^Department of Emergency Department, Second Xiangya Hospital, Central South University, Changsha, China; ^3^Emergency Medicine and Difficult Diseases Institute, Second Xiangya Hospital, Central South University, Changsha, China; ^4^College of nursing, Changsha Medical University, Changsha, Hunan, China

**Keywords:** type 2 diabetes mellitus, heart failure, BMI, waist circumference, T2DM

## Abstract

**Background:**

To determine the association of unintentional changes in body mass index (BMI) and waist circumference (WC) with the risk of heart failure (HF) among adults with type 2 diabetes mellitus (T2DM).

**Methods:**

This was a randomized controlled trial (the Action to Control Cardiovascular Risk in Diabetes [ACCORD] study), with a double 2×2 factorial design conducted at 77 clinical centers across the United States and Canada. In total, the study comprised 10,251 patients with T2DM and cardiovascular disease (CVD) or at a high risk of CVD. The outcome of interest in the present analysis was incident HF, defined as the first hospitalization event for HF or death due to HF. Hospitalization for HF was based on documented clinical and radiological evidence. Death due to HF was based on clinical, radiological, or postmortem evidence of HF, with an absence of an acute ischemic event according to clinical or postmortem evidence.

**Results:**

Participants with class III obesity had the smallest BMI and WC changes, followed by those with normal weight, overweight, class I obesity, and class II obesity. Increasing BMI (hazard ratio [HR] per standard deviation increase, 1.24; 95% confidence interval [CI], 1.07–1.45) and WC (1.27; 1.10–1.47) were significantly associated with a higher risk of HF. The relationship between BMI and WC changes and HF formed a J-shaped curve, while stable BMI and WC were associated with lower risks of HF. Compared with participants in the first tertiles of BMI and WC change, those in the third tertiles had HRs of 1.41 (95% CI, 1.07–1.45) and 1.48 (1.12–1.95), respectively.

**Conclusion:**

In conclusion, our findings suggest a noteworthy association between BMI and WC changes among adults with T2DM in HF. We observed a distinctive J-shaped curve in this relationship, indicating that participants with both low and high BMI and WC changes were more susceptible to developing HF.

**Trial registration:**

http://www.clinicaltrials.gov. Unique identifier: NCT00000620

## Background

The prevalence of obesity among adults in the US is projected to reach nearly 50% by 2030 (1). Obesity is a strong risk factor for developing type 2 diabetes mellitus (T2DM) and heart failure (HF) (2–4). Patients with T2DM tend to be overweight and have a high fat mass, which puts them at a high risk of HF. The prevalence of HF in patients with T2DM is up to four times higher than that in the general population (5). Given these premises, it is of utmost importance to focus on obesity in patients with T2DM to prevent the incidence of HF.

Numerous studies have identified obesity as a major risk factor for the development of HF (3, 6–8). A consensus statement from the International Atherosclerosis Society and International Chair on Cardiometabolic Risk Working Group on Visceral Obesity recommends that, in addition to body mass index (BMI) and body weight, waist circumference (WC) should be measured to assess cardiometabolic progression. Recent studies have revealed that WC, as a measure of abdominal obesity, is an indicator of body composition, and a large WC may pose a higher cardiovascular disease (CVD) risk, even in individuals with a normal BMI (9). In addition, in the Look AHEAD trial, a reduction in BMI and WC during follow-up was associated with a lower risk of HF among participants with T2DM who were overweight or obese (7, 10). Furthermore, bariatric surgery has been associated with a reduced risk of HF in over 5,000 patients with T2DM and obesity (11). Indeed, most patients with T2DM undergo unintentional weight changes, and the effects of unintentional BMI and WC changes on HF need to be elucidated (6). Unintentional weight loss is a sign of serious illness or other conditions, such as malignancy, trauma, incarceration, and starvation; thus, it must be addressed. Furthermore, till date, no study has been conducted in which the association between unintentional weight change and incident HF has been evaluated in patients with CVD or those with a high CVD risk and long history of T2DM. These patients tend to be overweight and have a higher fat mass and HF risk than do those without diabetes or those with new-onset T2DM (12, 13). Therefore, we evaluated the associations between unintentional changes in BMI and WC in patients with a 10-year history of T2DM and a high CVD risk. We hypothesized that increases in BMI and WC would be strongly associated with a higher risk of HF among patients with a 10-year history of T2DM.

## Methods

### Study population and data collection

The current study is a secondary analysis of data generated in a randomized controlled trial (the Action to Control Cardiovascular Risk in Diabetes [ACCORD] study) of 10,251 patients with T2DM and CVD or at a high CVD risk. The study, conducted at 77 clinical centers across the United States and Canada, employed a randomized, double 2×2 factorial design; it aimed to investigate the potential benefits of enhanced control over blood glucose, blood pressure, and/or lipid levels in improving cardiovascular outcomes among patients with T2DM. This trial’s design and main study outcomes have been previously published (14, 15). The average age of the participants with T2DM was 62 years, and all patients had a mean 10-year history of T2DM. After an average follow-up of 3.7 years, the intervention was discontinued because intensive glycemic control increased the risk of cardiac death, and all participants were transitioned to standard glycemic control and followed-up (**Supplementary**). Intensified blood and lipid control did not improve CVD outcomes during a median follow-up of 5 years. Participants with a history of HF were excluded from the present study. This included individuals with current symptomatic heart failure, a history of New York Heart Association (NYHA) class III or IV congestive heart failure at any point, or an ejection fraction (measured by any method) below 0.25.

### Exposure variables

Participants were followed-up annually, when data on anthropometric parameters, including WC and BMI, were collected. The median number of BMI and WC measurements was 5. Participants with fewer than three measurements of WC/BMI were excluded from the present analysis. **Supplementary Figure S1** illustrates the timing of the measurements of BMI and WC (measured annually). BMI and WC changes were defined as a linear trend (slope) in a participant’s BMI and WC during follow-up and were calculated as the change in BMI or WC per year. A positive value indicated a WC or BMI gain over time, whereas a negative value indicated a loss in WC or BMI. The definition of the exposure variables was used by many previously published articles (16, 17).

### Covariates and HF definition

Upon enrollment, the participants completed questionnaires and underwent physical examinations and laboratory measurements according to a standardized protocol, as previously described (16). The covariates assessed at baseline included age, sex, race, glycemic control strategy (intensive or standard), CVD history, educational status, depression status, smoking status, duration of diabetes, alcohol consumption, glomerular filtration rate, glycated hemoglobin (HbA1c), low-density lipoprotein (LDL), high-density lipoprotein (HDL), systolic blood pressure (SBP), and heart rate (HR). Smoking status was categorized as “never/former smoker” versus “current smoker” (last 30 days). Education levels were categorized as lower than high school, high school graduate, some college years, and college graduate or higher. Alcohol consumption was defined as weekly alcohol consumption.

The outcome of interest in the present analysis was incident HF, defined as the first hospitalization event for HF or death due to HF. In the ACCORD study, an independent committee adjudicated HF events as a prespecified outcome. Hospitalization for HF was based on documented clinical and radiological evidence. Death due to HF was based on clinical, radiological, or postmortem evidence of HF, with an absence of an acute ischemic event according to clinical or postmortem evidence. Time to event was calculated as the number of months until the occurrence of an HF event. Participants were censored at the time of their last follow-up.

### Statistical analysis

Continuous variables were compared using analyses of variance or Mann–Whitney U tests, and categorical variables were compared using chi-square analysis according to the distribution type. Missing data were coded as missing indicator categories for categorical variables, such as race, and using mean values for continuous variables, such as BMI and WC. Participants were divided into five groups based on their BMI: normal (BMI: 18.5 to < 25 kg/cm^2^), overweight (25 to < 30 kg/m^2^), class I obesity (BMI: 30 to < 35 kg/cm^2^), class II obesity (BMI: 35 to < 40 kg/cm^2^), and class III obesity (BMI: ≥ 40 kg/cm^2^). Using two models, we analyzed the relationship between BMI and WC changes and HF. Model 1 was adjusted for age, race, sex, glycemic control strategy (intensive or standard), education status, CVD history, duration of diabetes, cigarette, and alcohol consumption, and depression. Model 2 was further adjusted for LDL, HDL, GFR, HbA1c, SBP, and BMI/WC in addition to model 1. Tests for linear trends were conducted by treating quartile categories as continuous variables after assigning each category’s median value (18). Previous studies have demonstrated that weight change is associated with all-cause mortality (19, 20). We used restricted cubic splines with four knots at the 25th, 50th, and 75th percentiles to flexibly model the association of BMI and WC changes with HF adjusted for model 2. Subgroup and interaction analyses were performed according to age, sex, race, glycemic control strategy, smoking status, and CVD history.

All statistical analyses were two-sided, and we considered a P value < 0.05 statistically significant. All analyses were performed using Stata/MP software, version 17.0 (StataCorp LLC, College Station, TX).

### Patient and public involvement

We did not involve patients or the public in study design, implementation, interpretation of results, or development of communication strategies because this study was retrospective and used existing health system data. Our findings will also be shared on social media and related websites in the form of graphical and simplified summaries.

## Results

### Baseline characteristics of the included participants

**Figure 1:**
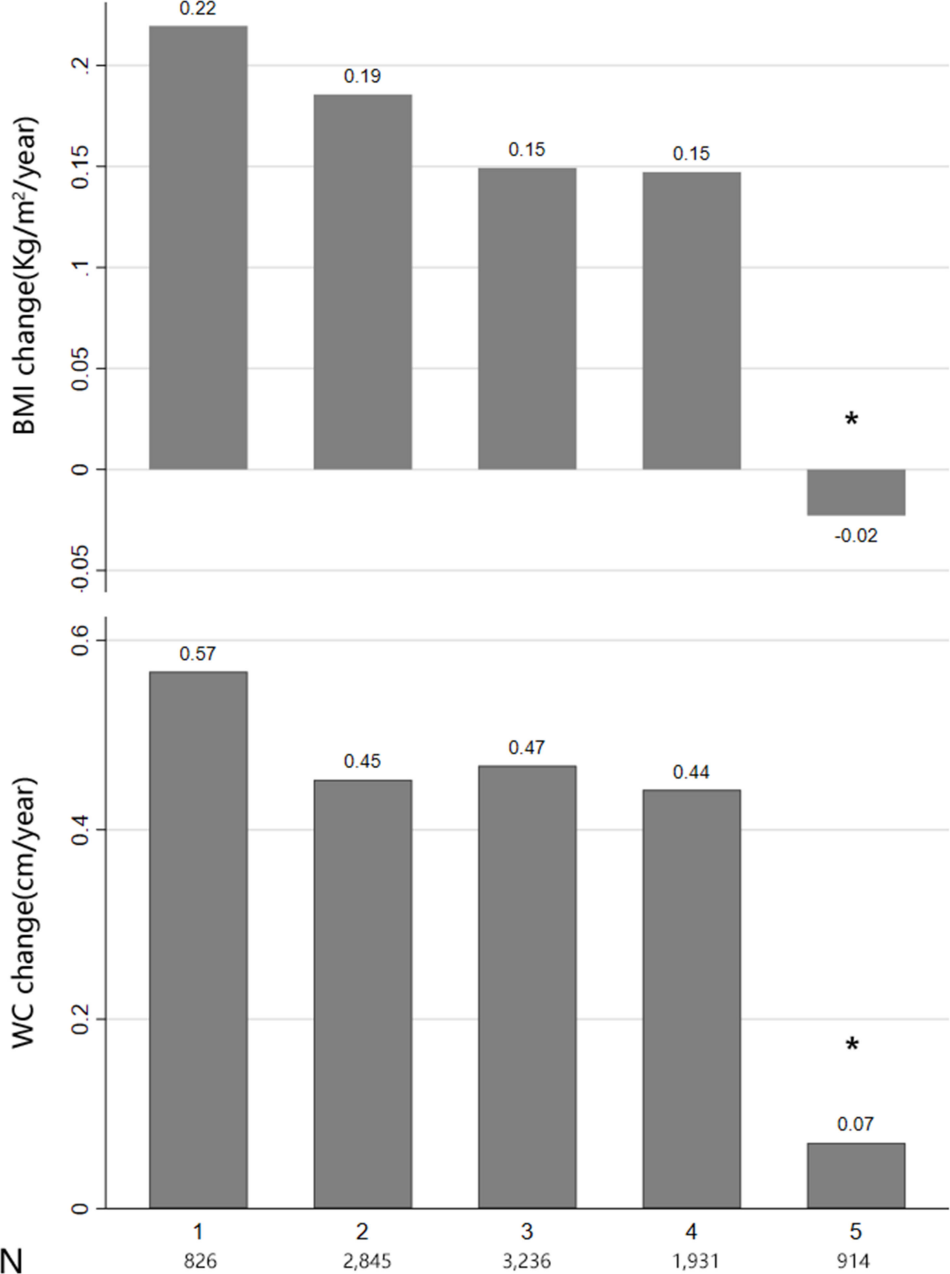
Body mass index (BMI) group and BMI/waist circumference change. Distribution of BMI groups based on World Health Organization classification: normal weight (18.5 ≤ BMI < 25), overweight (25 ≤ BMI < 30), class I obesity (30 ≤ BMI < 35), class II obesity (35 ≤ BMI < 40), and class III obesity (BMI ≥ 40 kg/m^2^).

Among the 10,251 participants enrolled in the ACCORD study, 494 were excluded from the present analyses for having HF before enrollment and 413 for having undergone less than three BMI/WC measurements. Hence, this study included 9,344 participants. The mean annual BMI change was 0.10 ± 0.48 kg/m^2^. The mean annual WC change was 0.05 ± 0.38 cm. The median number of BMI and WC measurements was 5 (interquartile range: 3–7) (**Supplementary Figure S2**). Participants with class III obesity had the smallest BMI/WC change (**Figure 1**). The baseline characteristics of the participants across tertiles of BMI and WC are summarized in **Table 1**. Participants with a BMI/WC increase were younger and had higher concentrations of urine protein, HbA1c, and LDL than did patients with a BMI/WC decrease. During a median follow-up of 5 (interquartile range: 4.1–5.7) years, 356 patients (cumulative rate: 3.7%) developed HF events; among these, 88 patients had an ischemic event.

**Table 1:** Characteristics stratified by BMI and WC change.

	Tertiles of BMI change (Kg/m^2^/year)			Tertiles of WC change(cm/year)		
	1(-10.0,-0.06)	2(-0.06,0.41)	3(0.41,6.0)	1 (-10.0, -0.25)	2(-0.25, 1.26)	3(1.26, 10.0)
**N**	3114	3116	3114	3081	3083	3083
**Age(years; mean ± SD)**	63.42 ± 6.66	62.81 ± 6.58	61.89 ± 6.36	63.19 ± 6.68	62.83 ± 6.56	62.05 ± 6.39
**Men**	1917 (61.56%)	1918 (61.55%)	1917 (61.56%)	1897 (61.57%)	1898 (61.56%)	1898 (61.56%)
Race						
**White**	1182 (37.96%)	1180 (37.87%)	1148 (36.87%)	1128 (36.61%)	1233 (39.99%)	1115 (36.17%)
**Non-White**	1932 (62.04%)	1936 (62.13%)	1966 (63.13%)	1953 (63.39%)	1850 (60.01%)	1968 (63.83%)
Glycemia control						
** Intensive**	1794 (57.61%)	1645 (52.79%)	1234 (39.63%)	1735 (56.31%)	1588 (51.51%)	1313 (42.59%)
** Standard**	1320 (42.39%)	1471 (47.21%)	1880 (60.37%)	1346 (43.69%)	1495 (48.49%)	1770 (57.41%)
**CVD history**	1022 (32.82%)	983 (31.55%)	1070 (34.36%)	1011 (32.81%)	1001 (32.47%)	1036 (33.60%)
Education						
**Less than high school**	475 (15.27%)	399 (12.81%)	472 (15.16%)	460 (14.94%)	435 (14.12%)	443 (14.37%)
**High-school graduate**	797 (25.62%)	786 (25.24%)	865 (27.78%)	793 (25.75%)	780 (25.32%)	849 (27.55%)
**Some college**	1048 (33.69%)	1032 (33.14%)	1006 (32.31%)	1019 (33.08%)	1042 (33.83%)	991 (32.15%)
**College degree or higher**	791 (25.43%)	897 (28.81%)	771 (24.76%)	808 (26.23%)	823 (26.72%)	799 (25.92%)
**Urine protein**	555 (17.82%)	599 (19.22%)	663 (21.29%)	588 (19.08%)	564 (18.29%)	638 (20.69%)
**Depression**	747 (23.99%)	677 (21.73%)	723 (23.23%)	769 (24.96%)	657 (21.31%)	702 (22.78%)
**Current smoker**	432 (13.87%)	399 (12.80%)	453 (14.55%)	431 (13.99%)	383 (12.42%)	458 (14.86%)
**Diabetes history (years; mean ± SD)**	10.62 ± 7.71	10.65 ± 7.51	10.85 ± 7.28	10.68 ± 7.71	10.61 ± 7.48	10.79 ± 7.27
**Alcohol/week (median, IQR; times)**	0, 0-0	0, 0-1	0, 0-0	0, 0-0	0, 0-1	0, 0-0
**BMI (kg/m^2^; mean ± SD)**	32.71 ± 5.33	31.19 ± 5.23	32.65 ± 5.43	32.60 ± 5.25	31.48 ± 5.47	32.44 ± 5.33
**WC (cm; mean ± SD)**	107.21 ± 13.46	104.27 ± 13.39	108.36 ± 13.45	109.62 ± 12.93	104.75 ± 13.93	105.49 ± 13.26
**HbA1c (%,; mean ± SD)**	8.17 ± 0.99	8.25 ± 1.02	8.46 ± 1.12	8.17 ± 0.98	8.28 ± 1.03	8.43 ± 1.12
**GFR**	91.27 ± 24.21	91.56 ± 30.63	91.62 ± 26.48	90.27 ± 25.53	92.57 ± 27.35	91.67 ± 28.84
Lipid (mg/dl; mean ± SD)						
** LDL**	104.59 ± 32.58	105.35 ± 33.76	104.89 ± 34.92	102.90 ± 32.65	105.68 ± 33.86	106.03 ± 34.55
** HDL**	41.91 ± 11.19	42.39 ± 11.70	41.70 ± 11.68	41.78 ± 11.11	42.21 ± 11.57	41.95 ± 11.87
**SBP (mmHg; mean ± SD)**	136.70 ± 16.92	136.16 ± 16.48	136.03 ± 17.01	135.97 ± 16.99	136.76 ± 16.22	136.20 ± 17.21
**DBP (mmHg; mean ± SD)**	75.19 ± 10.54	74.51 ± 10.36	75.38 ± 10.47	74.65 ± 10.49	75.03 ± 10.28	75.40 ± 10.63
**HR (beats/min; mean ± SD))**	71.97 ± 11.55	72.32 ± 11.62	73.71 ± 11.79	72.17 ± 11.65	72.45 ± 11.57	73.33 ± 11.77

CVD, cardiovascular disease; BMI, body mass index; WC, waist circumference; HbA1c: glycated hemoglobin; GFR, glomerular filtration rate; LDL, Low density lipoprotein; HDL, high density lipoprotein; SBP, systolic blood pressure; DBP, diastolic blood pressure; HR, heart rate; IQR, inter quartile range.

### Relationship between BMI and WC changes and HF

The association between BMI and WC changes and the risk of HF is presented in **Table 2**. In the adjusted analysis, compared with the first tertile, the risk of HF was relatively lower in the second tertile (BMI change: hazard ratio, 0.82; [95% confidence interval, 0.61–1.10]; WC change: 0.91 [0.67–1.22]) after adjusting for confounding variables in model 2. However, patients in the third tertile had a higher risk of HF (BMI change: 1.41 [1.07–1.84]; WC change: 1.48 [1.12–1.95]) than did those in the first tertile in the adjusted analysis (model 2). When we treated BMI and WC changes as continuous variables, the per-SD increase in BMI and WC was associated with a 24% and 27% higher risk of HF, respectively (BMI change: 1.24 [1.07–1.45]; WC change: 1.27 [1.10–1.47]).

**Table 2:** The association of tertiles of BMI/WC change and HF risk.

	Incidence rate per 1000 person years	Model 1	Model 2
BMI change(kg/m^2^/year)			$
1 (-10.0, -0.06)	7.6	Ref	Ref
2 (-0.06, 0.41)	5.2	0.75 (0.56,0.99)	0.82 (0.61,1.10)
3 (0.41, 6.0)	9.7	1.41 (1.09, 1.81)	1.41 (1.07,1.84)
HR per SD*		1.24 (1.08-1.43)	1.24 (1.07-1.45)
P for trend		0.01	<0.01
WC change (cm/year)			#
1 (-10.0, -0.25)	7.7	Ref	Ref
2 (-0.25, 1.26)	5.7	0.76 (0.57,1.00)	0.91 (0.67,1.22)
3 (1.26, 10.0)	9.1	1.27 (0.98,1.64)	1.48 (1.12,1.95)
HR per SD*		1.14 (1.01-1.28)	1.27 (1.10-1.47)
P for trend		0.08	<0.01

Model 1: age, race, sex, glycemic control strategy (intensive or standard) education status, CVD history, duration of diabetes, cigarette, and alcohol consumption.

Model 2: model 2 in addition to LDL, GFR, HDL, HbA1c, SBP, BMI/WC; $ adjusted for baseline BMI, # adjusted for baseline WC, *The SD is the standard deviation of BMI or WC change per year.

**Figure 2:**
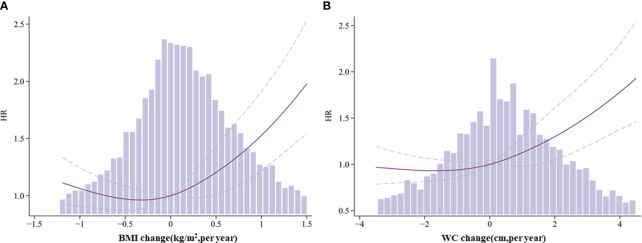
Association between body mass index (BMI)/waist circumference (WC) change and HF among type 2 diabetes mellitus patients. **(A)** BMI change and heart failure (HF). **(B)** WC change and HF. Solid lines represent hazard ratios (HR) and the dummy line indicates the 95% confidence interval (CI). HR values are adjusted for Model 2. Blue columns represent the change in BMI or WC per year.

We visualized the non-linear association of BMI/WC changes and HF incidence by using restricted cubic splines for flexible modeling, as demonstrated in **Figure 2**. A J-shaped relationship was observed between BMI/WC changes and HF (P < 0.01). The HF risk was relatively flat or decreased slowly until the BMI or WC change reached approximately 0, following which it increased rapidly.

### Subgroup analysis

**Figure 3:**
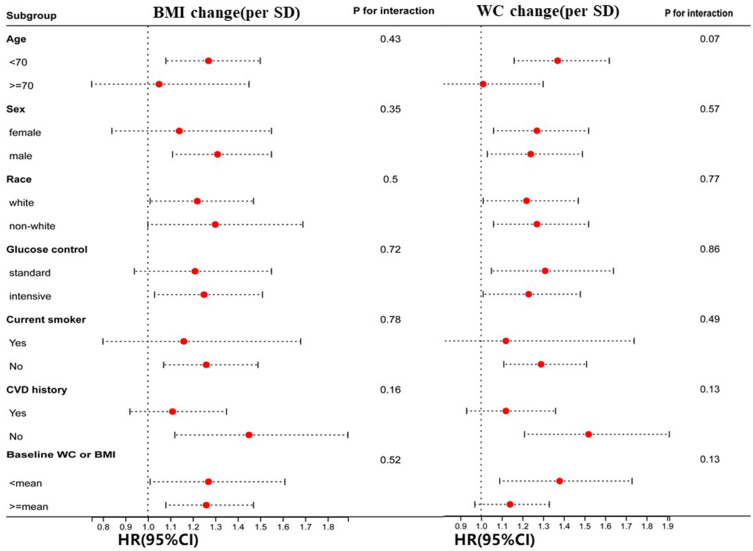
Hazard ratios per one standard deviation (SD) increase in body mass index (BMI)/waist circumference (WC) change for heart failure (HF). Hazard ratios per one SD increase in BMI/WC change for the occurrence of HF. Each stratification is adjusted for all factors in Model 2.

The association between BMI/WC change and incident HF in different subgroups is illustrated in **Figure 3**. The results revealed that none of the factors (age, sex, race, glycemic control strategy, smoking status, and CVD history) played an interactive role in the association between BMI/WC change and HF incidence.

## Discussion

This *post hoc* analysis documented that WC and BMI changes are independent risk factors of HF among participants with T2DM and CVD or at a high risk of CVD, displaying a J-shaped curve. After various degrees of adjustment, individuals who had an increase in BMI and WC had a higher incidence of HF, even after adjusting for WC and BMI, respectively. An equally interesting observation is that participants with a baseline normal weight and overweight exhibited greater BMI and WC increase; such individuals should especially focus on keeping their weight and WC stable, as it may reduce their incidence of HF. Taken together, monitoring WC and BMI changes in patients with T2DM and CVD or at a high CVD risk may be important in tracking the incidence of HF.

Patients with T2DM tend to be overweight and have a higher fat mass, WC, and HF risk than do those without diabetes (12, 13). The Look AHEAD trial demonstrated that intentional WC change was significantly associated with HF among patients with new-onset T2DM who were overweight or obese (7). However, in most patients with T2DM, BMI and WC changes are unintentional. Our present study suggested that both weight and WC loss and gain are associated with a higher HF incidence, which differs from studies that reported that BMI loss and WC decline were associated with a lower risk of HF (7). Furthermore, participants had a higher risk of HF than did participants who underwent a decline in BMI/WC. In contrast with prior studies, this study was mainly focused on patients with T2DM with unintentional BMI/WC changes and CVD or a high CVD risk. Furthermore, the participants in this study were older and had a longer history of diabetes than did those in the Look AHEAD trial.

There are several potential biological mechanisms underlying the relationship between adiposity and the risk of HF. Weight gain increases the risk of atherosclerosis, which is the primary mechanism underlying the development of HF (21). Central adiposity, assessed via WC, is related to cardiac remodeling patterns, leading to HF development (22). The increased deposition of lipids can be lipotoxic and induce apoptosis of cardiomyocytes, non-hypertensive cardiomyopathy, left ventricular hypertrophy and dysfunction, and insulin resistance, all of which are associated with an increased risk of HF (23, 24). Additionally, BMI and WC loss due to muscle and fat wasting also provide a possible link to the development of HF (25). Shah et al. reported that weight loss was related to left ventricular concentricity and mass (26). Furthermore, underlying diseases in older individuals and those with poor nutritional status cause many changes in body composition, often accompanied by changes in body weight and BMI. In particular, aging leads to a decrease in metabolically active muscle mass, resulting in inefficient oxygen extraction, which increases the risk of HF (27).

Our study has several advantages. First, it is, to our knowledge, the first large-scale study with a long follow-up period focused on unintentional weight change and incident HF among patients with T2DM. The sample was sufficiently large to evaluate the association and conclusively examine sex and race interactions. We also used different indicators of obesity to verify the robustness of our findings. Second, in previous cohort studies, the dynamic features of body weight over time were overlooked, and weight was measured only twice per participant (20). Weight loss is frequently followed by weight gain and other weight fluctuation patterns (2). In the present study, we measured weight and WC in the entire ACCORD study cohort, and each participant’s weight and WC were measured at least three times.

Our results have several important clinical implications. In this observational study, unintentional increases in BMI and WC were positively associated with the incidence of HF among patients with T2DM. These observations underscore the need for this high-risk population to avoid excessive weight and WC fluctuations, particularly in cases of normal weight or overweight. Additional studies are needed to evaluate whether sustained weight loss and WC reduction are associated with the risk of HF.

The present study had several limitations. First, our predefined outcomes did not distinguish between HF with a reduced ejection fraction (EF) and HF with a preserved EF. The effects of BMI and WC on HF may differ among HF subtypes. Second, it is difficult to establish causal relationships due to the nature of the study design. Third, the use of drugs that may be effective for HF, such as SGLT2 inhibitors and GLP-1 receptor agonists, was underrepresented because recruitment in the ACCORD study ceased in 2005. Therefore, adjustments for such factors were not made in the analysis. Fourth, all included patients were from the US and Canada, and these results may not apply to other populations (e.g., Asian populations) that exhibit different characteristics and lifestyles. Fifth, residual confounding cannot be averted in observational studies. However, we believe that residual confounding was a minor issue in the present study, as we adjusted for all known relevant confounders in HF incidence. Furthermore, the adjustments had little effect on the effect size.

## Conclusion

In conclusion, our findings suggest a noteworthy association between BMI and WC changes among adults with T2DM in HF. We observed a distinctive J-shaped curve in this relationship, indicating that participants with both low and high BMI and WC changes were more susceptible to developing HF. Notably, an increase in weight emerged as a significant factor associated with a higher likelihood of HF development in this population.

## Availability of data and materials

Data are available from the Biologic Specimen and Data Repository Information Coordinating Center (BioLINCC).

## Ethics approval and consent to participate

This is a *post-hoc* analysis of the ACCORD study. No participants were involved in setting the research questions, outcome measures, or in the design and implementation of the study. No plans exist to involve patients in the dissemination.

## Data availability statement

The raw data supporting the conclusions of this article will be made available by the authors, without undue reservation.

## Author contributions

YZ: Data curation, Writing – original draft. XC: Conceptualization, Supervision, Writing – review & editing. GY: Software, Writing – original draft. XS: Data curation, Writing – original draft. ZX: Conceptualization, Funding acquisition, Writing – review & editing.
