# Supplementary material for: Changes in body mass index and waist circumference and heart failure in type 2 diabetes mellitus
Source: Front Endocrinol (Lausanne). 2023 Dec 21;14:1305839. doi: 10.3389/fendo.2023.1305839 (PMC10764620; doi:10.3389/fendo.2023.1305839)
Supplement: Supplementary file 1 [file DataSheet_1.docx]

**Changes in body mass index and waist circumference and heart failure in type 2 diabetes mellitus**

Yang Zhou^1^, Xiangping Chai^2,3^, Guifang Yang^2,3^, Xin Sun^4^, Zhenhua Xing^2,3*^

1. Department of Intensive Care Unit, Second Xiangya Hospital, Central South University, Changsha 410011, China

2. Department of Emergency Department, Second Xiangya Hospital, Central South University, Changsha 410011, China

3. Emergency Medicine and Difficult Diseases Institute, Second Xiangya Hospital, Central South University, Changsha 410011, China.

4. College of nursing, Changsha Medical University, Changsha, Hunan province, 410000, China

*Corresponding author: Zhenhua Xing, MD

E-mail: xing2012x@csu.edu.cn

Phone number: +8615084714930

Supplementary

In this trial, participants will be randomized to one of two treatment groups based on the targeted level of glycemic control. Both the intensive and standard therapy groups will utilize all currently available glucose-lowering therapies. The two treatment groups will have different glycemic targets and will have different thresholds of glycemic control at which therapeutic changes will be considered (Table 3.1).


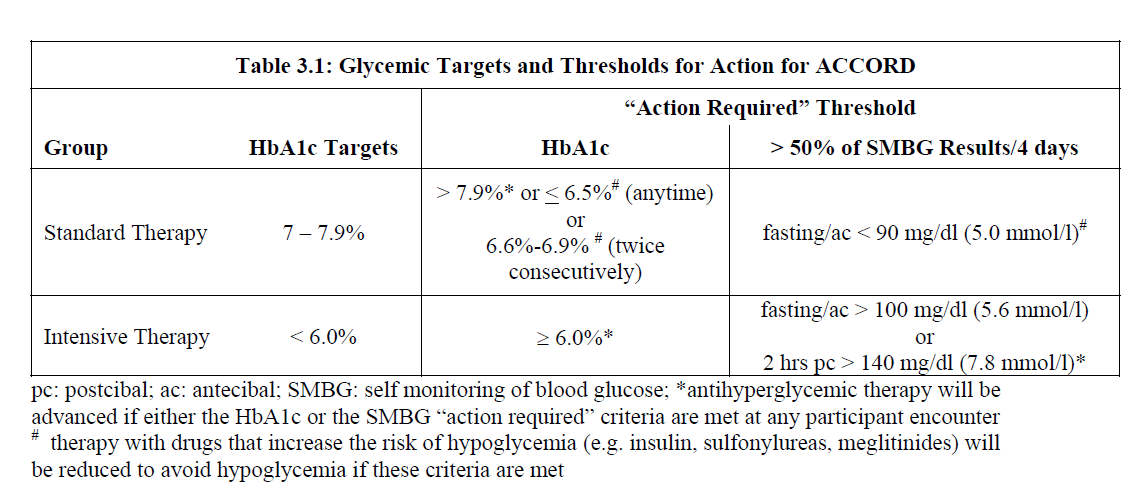


To achieve these glycemic targets, participants will require self-management education and dietary and lifestyle interventions, as well as pharmacologic therapy. They will also require different drug choices and treatment intensities.

**
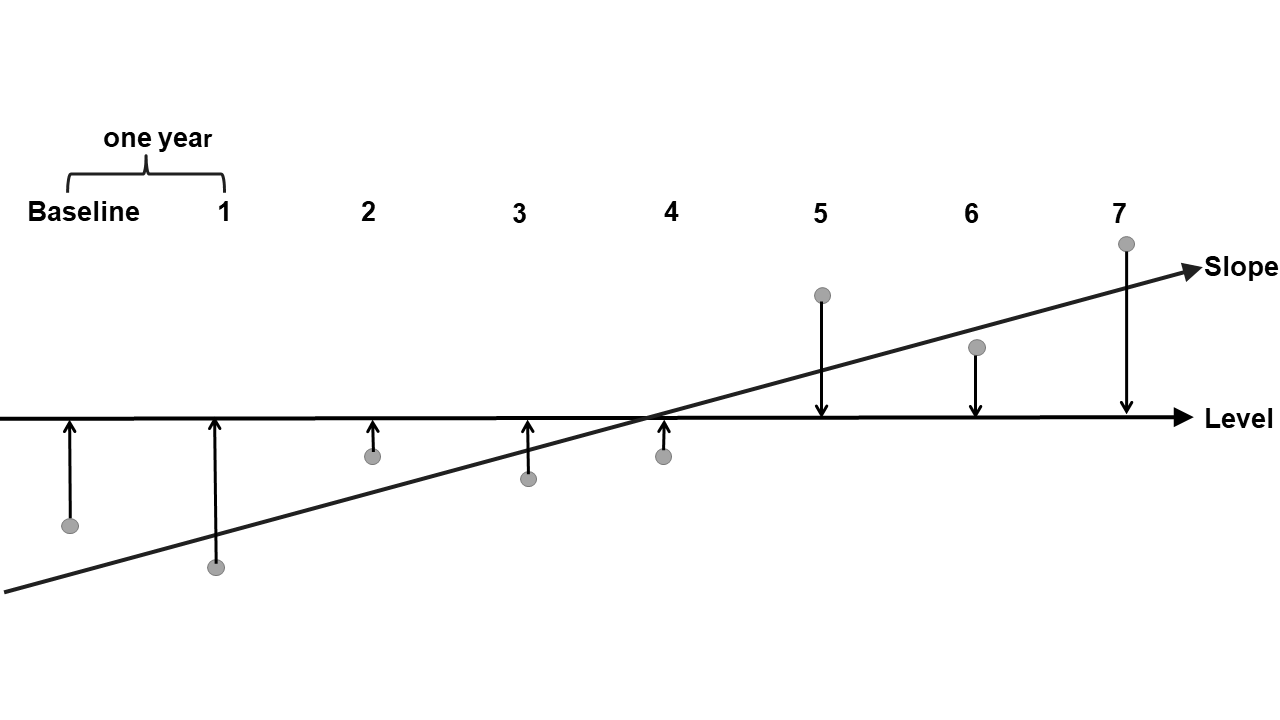
**

**sFig 1: Time interval and schematic representation of BMI/ WC change for a hypothetical subject.**

Patients were followed every year, a regression between WC/BMI change and follow-up time points was performed. Regression coefficient represents BMI change or WC change per year(slope). Levels: mean BMI/WC of a subject; the linear trend in BMI/WC per year (slope)^[1]^


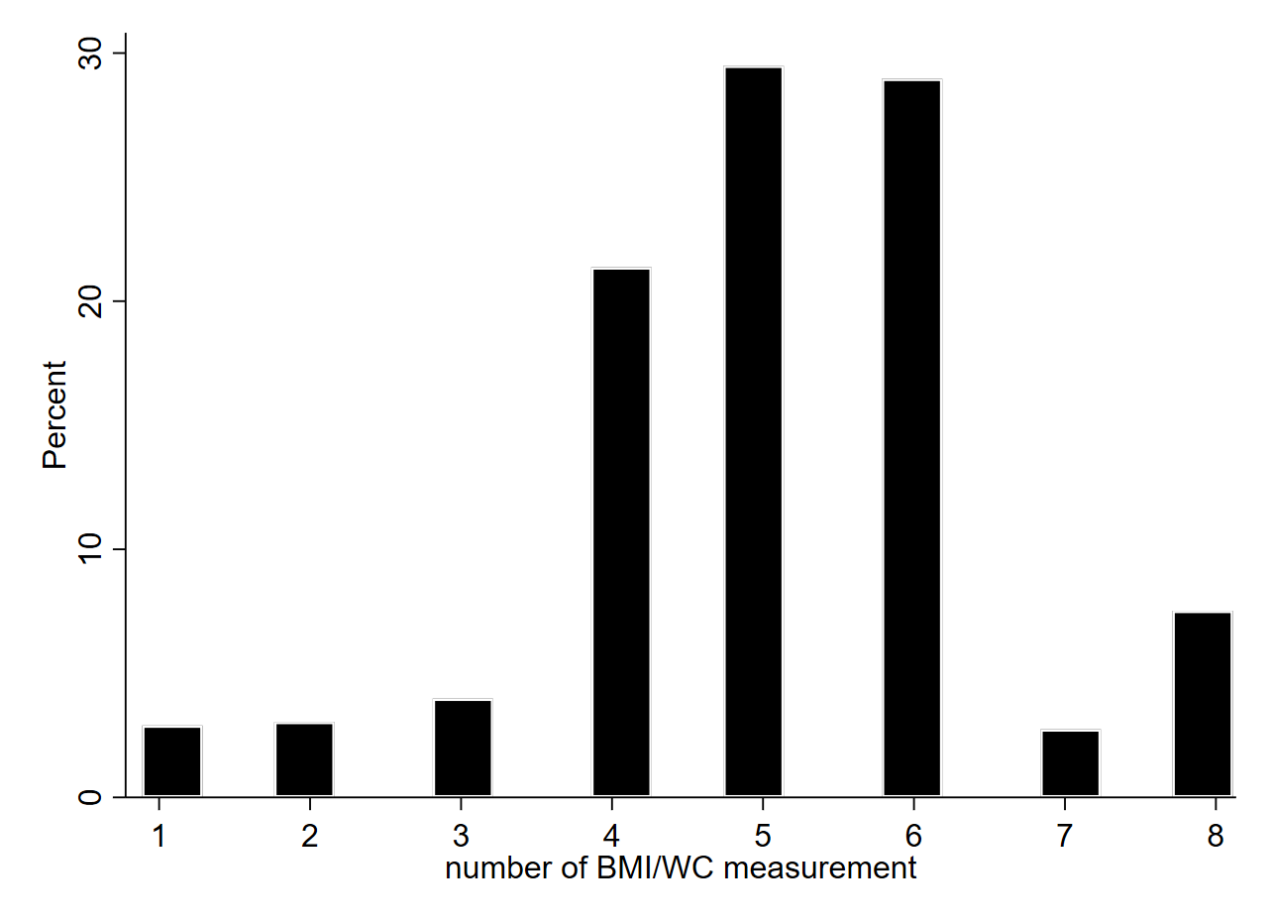


**sFig 2: number of BMI/WC measurements druing follow-up period**

**References**

[1] Xing Z, Chai X. Changes in fat mass and lean body mass and outcomes in type 2 diabetes mellitus. Intern Emerg Med. 2022 .
